# Adenylosuccinate lyase deficiency

**DOI:** 10.1007/s10545-014-9755-y

**Published:** 2014-08-12

**Authors:** Agnieszka Jurecka, Marie Zikanova, Stanislav Kmoch, Anna Tylki-Szymańska

**Affiliations:** 1 Department of Genetics, University of Gdańsk, ul. Wita Stwosza 59, 80-308 Gdańsk, Poland; 2 Institute of Inherited Metabolic Disorders, First Faculty of Medicine, Charles University, General University Hospital, Prague, Czech Republic; 3 Department of Pediatrics, Nutrition and Metabolic Diseases, The Children’s Memorial Health Institute, Warsaw, Poland

## Abstract

Adenylosuccinate lyase ADSL) deficiency is a defect of purine metabolism affecting purinosome assembly and reducing metabolite fluxes through purine *de novo* synthesis and purine nucleotide recycling pathways. Biochemically this defect manifests by the presence in the biologic fluids of two dephosphorylated substrates of ADSL enzyme: succinylaminoimidazole carboxamide riboside (SAICAr) and succinyladenosine (S-Ado). More than 80 individuals with ADSL deficiency have been identified, but incidence of the disease remains unknown. The disorder shows a wide spectrum of symptoms from slowly to rapidly progressing forms. The fatal neonatal form has onset from birth and presents with fatal neonatal encephalopathy with a lack of spontaneous movement, respiratory failure, and intractable seizures resulting in early death within the first weeks of life. Patients with type I (severe form) present with a purely neurologic clinical picture characterized by severe psychomotor retardation, microcephaly, early onset of seizures, and autistic features. A more slowly progressing form has also been described (type II, moderate or mild form), as having later onset, usually within the first years of life, slight to moderate psychomotor retardation and transient contact disturbances. Diagnosis is facilitated by demonstration of SAICAr and S-Ado in extracellular fluids such as plasma, cerebrospinal fluid and/or followed by genomic and/or cDNA sequencing and characterization of mutant proteins. Over 50 ADSL mutations have been identified and their effects on protein biogenesis, structural stability and activity as well as on purinosome assembly were characterized. To date there is no specific and effective therapy for ADSL deficiency.

## Definition

Adenylosuccinate lyase deficiency (OMIM 103 050) is an autosomal recessive defect of purine metabolism. It was first described in 1984 by Jaeken and van den Berghe (Jaeken and Van den Berghe 1984), who found succinylpurines in the cerebrospinal fluid (CSF), plasma, and urine of three patients with severe psychomotor delay and autistic features.

**Fig. 1:**
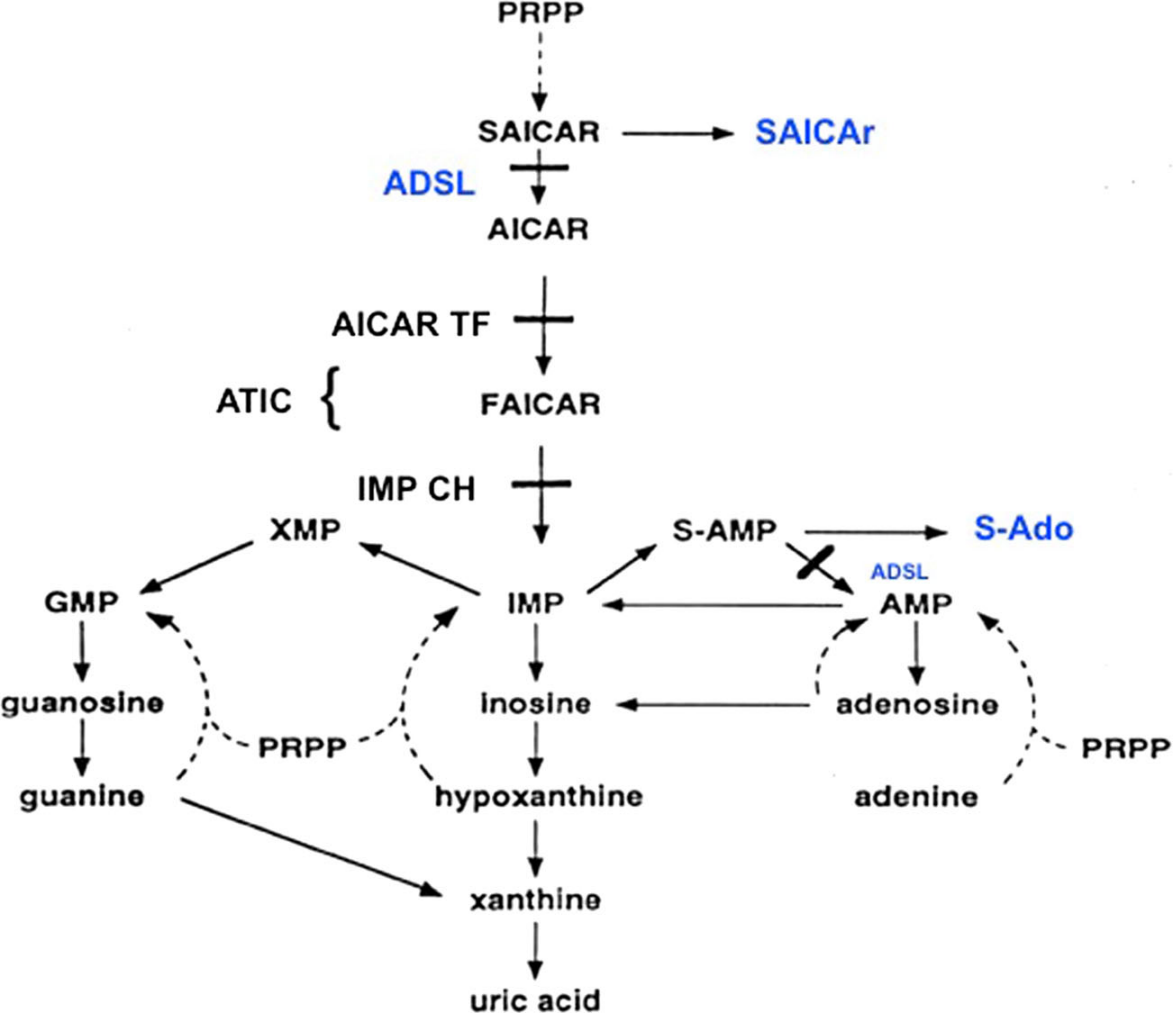
*De novo* purine biosynthesis pathway. Abbreviations are as follows: PRPP, phosphoribosylpyrophosphate; SAICAR, succinylaminoimidazole carboxamide ribotide; SAICAr, succinylaminoimidazole carboxamide riboside; AICAR, aminoimidazole carbozamide ribotide; FAICAR, formyloaminoimidazole carboxamide ribotide; IMP, inosine monophosphate; *S*-AMP, adenylosuccinate; *S*-Ado, succinyladenosine; AMP, adenine monophosphate; XMP, xanthine monophosphate; GMP, guanine monophosphate; ADSL, adenylosuccinate lyase; AICAR TF, aminoimidazole carboxamide riboside transformylase; IMP CH, inosine monophosphate cyclohydrolase; ATIC, bifunctional enzyme AICAR transformylase/IMP cyclohydrolase

The enzyme adenylosuccinate lyase (EC 4.3.2.2) catalyzes two non-sequential steps in the *de novo* synthesis of purine nucleotides leading to nonhydrolytic cleavage of succinyl groups to yield fumarate: the conversion of succinylaminoimidazole carboxamide ribotide (SAICAR) into aminoimidazole carboxamide ribotide (AICAR) and the formation of adenosine monophosphate nucleotides (Fig. 1). In addition to its role in purine biosynthesis, the enzyme is involved, together with adenylate deaminase and adenylosuccinate synthetase, in the purine nucleotide cycle. This cycle prevents adenosine monophosphate (AMP) accumulation following adenosine triphosphate (ATP) catabolism, and this, in turn, displaces the adenylate kinase reaction toward adenosine triphosphate formation (Sabina et al 1980). Moreover, the purine nucleotide cycle plays a role in maintaining the adenylate energy charge through the generation of intermediates for the citric-acid cycle from amino acids and through the stimulation of activities of glycogen phosphorylase and of phosphofructokinase, thus enhancing the rate of glycolysis (Tornheim and Lowenstein 1975; Aragon and Lowenstein 1980; Young et al 1996).

The native human ADSL protein is a homotetramer with a subunit size of ~50 kDa. The protein contains four active sites. Each of them is formed from regions of three different subunits - Protein Data Bank (PDB) (2VD6; http://www.pdb.org/pdb/cgi/explore.cgi?pdbId=2VD6).

The enzyme defect (MIM 103050) manifests itself by the presence in the biologic fluids of two normally undetectable compounds, succinylaminoimidazole carboxamide riboside (SAICAr) and succinyladenosine (S-Ado), which are formed by dephosphorylation of the two substrates of the enzyme.

## Epidemiology

Incidence of ADSL deficiency remains unknown. To date almost 80 patients have been reported and the majority of them were found in Holland and Belgium, where the defect was discovered for the first time (Jaeken and Van den Berghe 1984; Kohler et al 1999). Other reported patients were from Czech Republic (Kmoch et al 2000; Mouchegh et al 2007), Poland (Jurecka et al 2008a, b), Germany (Kohler et al 1999; Mouchegh et al 2007), UK (Lundy et al 2010), Spain (Castro et al 2002; Perez-Duenas et al 2011), Italy (Nassogne et al 2000; Race et al 2000), Portugal (Edery et al 2003), France (Holder-Espinasse et al 2002), Norway (Marie et al 2002), Turkey (Kmoch et al 2000; Spiegel et al 2006), Morocco (Jaeken and Van den Berghe 1984; Stone et al 1992; Gitiaux et al 2009), Malaysia (Chen et al 2010), Australia (Stathis et al 2000; van Werkhoven et al 2013), Colombia (Castro et al 2002), and the US (Kmoch et al 2000; Spiegel et al 2006). Detailed and regularly updated information on identified patients may be found on ADSL database http://www1.lf1.cuni.cz/udmp/adsl.

## Clinical description

### Clinical heterogeneity

Although there is wide variation in the clinical presentation observed in patients with ADSL deficiency, descriptive systems have classified patients’ phenotypes as severe type I form, milder type II form, and fatal neonatal form (Jurecka et al 2008a, b).

#### Fatal neonatal form of ADSL deficiency

Recently, a larger number of patients with a neonatal form have been reported (van den Bergh et al 1998; Jurkiewicz et al 2007; Mouchegh et al 2007; Jurecka et al 2008a, b; Lundy et al 2010). These patients presented with fatal neonatal encephalopathy with a lack of spontaneous movement (“floppy infant”), respiratory failure and intractable seizures resulting in early death within the first weeks of life. Additionally it has been reported that ADSL may have prenatal manifestations, with impaired intrauterine growth, microcephaly, fetal hypokinesia (with all its consequences, up to arthrogryposis and pulmonary hypoplasia), and a loss of fetal heart rate variability (Mouchegh et al 2007).

#### Type I ADSL deficiency

Most of the patients reported so far have adenylosuccinate lyase deficiency type I (severe form) with a purely neurologic clinical picture characterized by severe psychomotor retardation, early onset of seizures and microcephaly. These patients present within the first months of life and the further evolution is characterized by developmental arrest, lack of eye-to-eye contact and in some patients coma vigil (Jaeken et al 1988; Krijt et al 1994; Valik et al 1997; Kohler et al 1999; Jurecka et al 2008a, b; Gitiaux et al 2009; Lundy et al 2010; Jurecka et al 2012a, b). Severe cortical visual impairment corresponding to the degree of encephalopathy may also be a feature of the severe phenotype (Lundy et al 2010).

#### Type II ADSL deficiency

Patients with type II (moderate or mild form), who develop symptoms within the first years of life, usually suffer from slight to moderate psychomotor retardation and transient contact disturbances (Jaeken et al 1988; Jaeken et al 1992; Jurecka et al 2008a, b; Jurecka et al 2014). Seizures, if present, appear later, often between the 2nd and 4th year of life (Castro et al 2002; Jurecka et al 2008a, b), but also reported starting as late as the 9th year of life (Gitiaux et al 2009). Speech impairment with a minimal use of words was noticed that contrast with a higher degree of receptive and non-verbal communication skills (Gitiaux et al 2009). Another described problem is ataxia, which may be the cause of increasing gait disturbance.

The disease manifests symptoms along a continuum and despite the utility of communicating with the above-mentioned three descriptive categories; there are no fixed parameters to ascribe a particular patient to a single category. It has been suggested that the ratio of the accumulating succinyladenosine (S-Ado) and succinylaminoimidazolecarboxamide riboside (SAICAr) in body fluids is not predictive of phenotype severity; rather, it may be secondary to the degree of the patient’s development (i.e., to the age of the patient at the time of a sample collection) (Zikanova et al 2010).

### Clinical manifestations

The majority of ADSL-deficient children are born after uncomplicated pregnancies with normal birth and family history. The neonatal period in mild form might be normal with growth parameters in the normal range. In severe type of disease neurologic symptom might occur early after birth (Ciardo et al 2001).

Neurological symptoms are the most common and prominent clinical problems associated with adenylosuccinate lyase deficiency. Particularly common neurologic presentations include acute encephalopathy, chronic encephalopathy, and behavioral abnormalities.

#### Acute encephalopathy (with seizures)

Acute encephalopathy is the most common presentation in the neonate and, typically, the affected neonate is born after a normal pregnancy, at or near term, with a normal birth weight and remains well in the early hours or days of life. Such an infant may be discharged from the newborn nursery, subsequently presenting as acutely unwell. Early signs of encephalopathy are non-specific, such as poor feeding, lethargy, vomiting, abnormalities of tone, and irritability. Later problems may include drowsiness, fits, hiccups, myoclonus, apneic episodes, marked hypotonia, irritability with cycling movements, seizures, and coma. Cerebral oedema may develop, contributing to the relentless deterioration if not treated.

ADSL deficiency can cause onset of epilepsy in the first year of life. Seizures in early infancy should be thoroughly investigated for a metabolic basis while other, more common, causes are investigated. The clinical picture is usually complex and epilepsy is one of various other associated neurological symptoms, such as mental retardation, hypotonia, and microcephaly. Importantly, convulsions associated with ADSL deficiency may have a very early onset and be the reason for which the infant is referred to a neurologist in the first months of life, despite the presence of other, less easily identified symptoms such as delayed psychomotor acquisitions or muscle tone disorders. General characteristics of seizures secondary to ADSL deficiency are as follows: onset in the first months of life, usually partial; simple partial motor semiology of brief duration; and successive appearance, often closely related to partial seizures, tonic seizures, spasms, and massive myoclonus. Resistance to treatment is common.

#### Chronic encephalopathy (with or without seizures)

Developmental delay and psychomotor retardation are common in adenylosuccinase deficiency. There are some characteristics of the cognition which, when present should alert the clinician to the possibility of an underlying ADSL defect. First, it tends to be global, affecting all spheres of development to some extent. Although a mild developmental problem may present as speech delay, in most cases, a careful history and developmental examination shows that the defect extends to other developmental spheres. Secondly, severe irritability, impulsivity, aggressiveness, and hyperactivity are also common among infants with mental retardation caused by PP defects. Thirdly, the psychomotor retardation is usually progressive. There is generally a history of a period of apparently normal development, followed by loss of developmental milestones or progressive deterioration in school performance. Fourthly, the psychomotor retardation is usually associated with other objective evidence of neurologic dysfunction, such as disorders of tone (hypotonia sometimes followed after years by spasticity), seizures, pyramidal tract signs or evidence of extrapyramidal deficits.

### Seizures

Corresponding to the clinical diversity, the epileptic phenotypes associated with ADSL deficiency are also highly variable, comprising myoclonus, partial seizures, infantile spasms, and status epilepticus (Ciardo et al 2001; Lundy et al 2010). Seizures usually start either early in the neonatal period or after the first year of life (van den Bergh et al 1998; Jurecka et al 2012a, b). Approximately half of the patients with ADSL deficiency suffer from epilepsy, which is often intractable, but not always associated with status epilepticus (Ciardo et al 2001).

#### Behavioral abnormalities

The autistic features in patients with ADSL deficiency include failure to make eye-to-eye contact, repetitive behavior, agitation, temper tantrums, and autoaggressivity (Van den Berghe et al 1997). Several stereotypies might include hand movements, repetitive manipulation of toys, grimacing, clapping hands, rubbing feet, inappropriate laughter, head and trunk rocking, and stereotyped sounds (Ciardo et al 2001; Perez-Duenas et al 2011). Gitiaux et al reported behavioral features in two patients, which combined excessive laughter, a very happy disposition, hyperactivity, a short/lack attention span, the mouthing of objects, tantrums, and stereotyped movements (Gitiaux et al 2009).

#### Dysmorphic features

ADSL deficiency has been reported to have some subtle dysmorphisms, but the primary and the most obvious problem is the biochemical derangement. Microcephaly is a characteristic clinical feature, but is not invariably present in the most mildly affected patients (Lundy et al 2010). Brachycephaly, flat occiput, prominent metopic sutures, intermittent divergent strabismus, small nose with anteverted nostrils, long, smooth philtrum, and thin upper lip and low set ears have been reported by Holder-Espinasse (Holder-Espinasse et al 2002). Other authors described wide mouth, wide-spaced teeth and a prominent mandible (Gitiaux et al 2009).

#### Other features

Hypotonia (axial and generalized) is a common feature of severe type of ADSL deficiency and is often combined with peripheral hypertonia.

### Magnetic resonance imaging studies

**Fig. 2:**
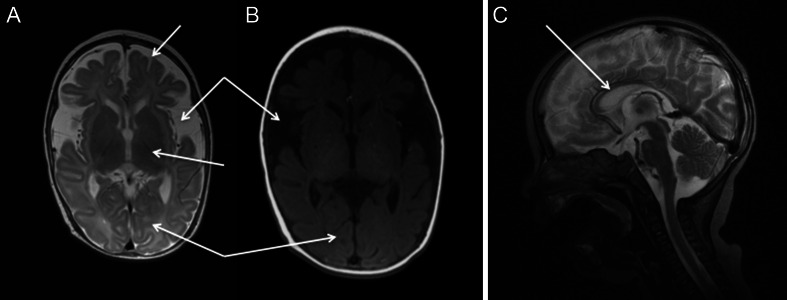
**a**-**c**. Axial and sagittal T_2_-weighted and T_1_-weighted cranial magnetic resonance imaging of a patient with type I ADSL deficiency: arrows indicate myelination of the posterior limbs of the internal capsules, enlarged subarachnoid space in the frontal regions, widened lateral ventricles (especially enlargement of the body and occipital horns), wide Sylvian fissures with hypoplasia operculum, thin corpus callosum and lack of myelination of the white matter volume

**Fig. 3:**
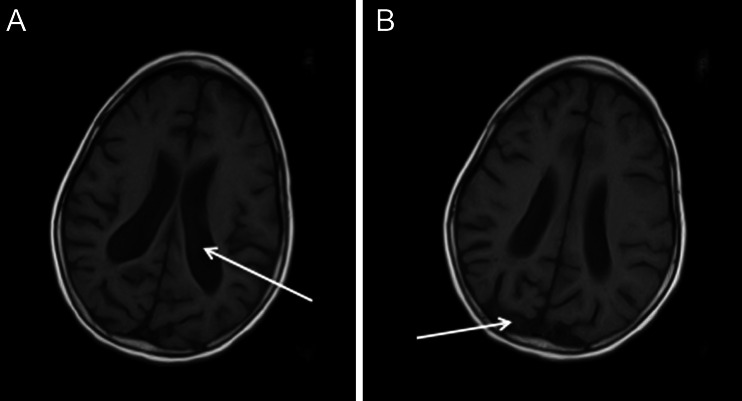
**a**-**b**. Axial T_2_-weighted and T_1_-weighted cranial magnetic resonance of a patient with type I ADSL deficiency: arrows indicate symmetrical widening of the lateral ventricles, enlarged sulci of the brain cortex gyri, especially in the parieto-occipital lobes

**Fig. 4:**
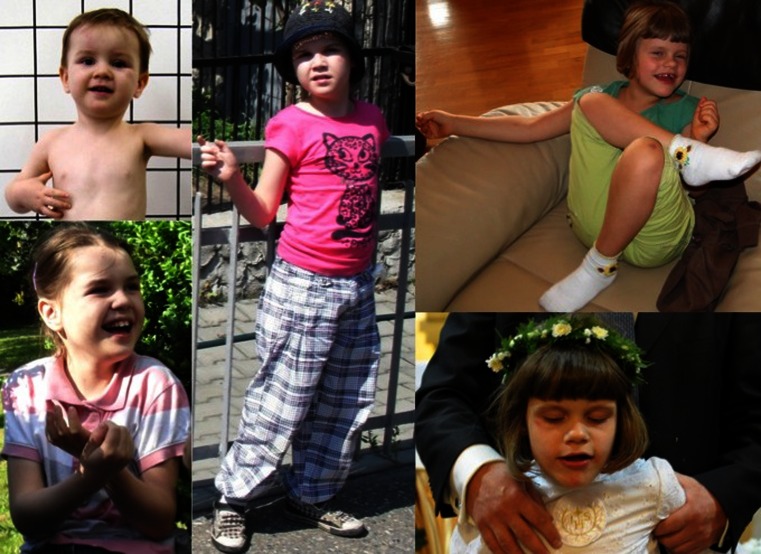
Photos of patients with type II adenylosuccinate lyase deficiency

Magnetic resonance imaging (MRI) can be a useful tool for diagnosing and monitoring the pathological progression of the changes in the course of the disease. Although over 60 patients with adenylosuccinate lyase deficiency have been reported, there are only 12 reports of brain imaging findings associated with the condition. These are mostly case reports or small series of cases with different phenotypes, and correlation with clinical symptoms is ambiguous. Reported findings on brain MRI in ADSL deficiency include atrophy of the cerebral cortex, corpus callosum, cerebellar vermis (Van den Berghe et al 1997; Edery et al 2003; Jurecka et al 2012a, b), lack of myelination (Kohler et al 1999; Jurecka et al 2012a, b), delayed myelination (Jurkiewicz et al 2007; Lundy et al 2010; Jurecka et al 2012a, b), anomalies of the white matter (Valik et al 1997; Nassogne et al 2000; Edery et al 2003; Marinaki et al 2004; Lundy et al 2010) and lissencephaly (Stathis et al 2000) (Figs. 2, 3 and 4).

## Etiopathogenesis

Adenylosuccinate lyase deficiency is inherited as an autosomal recessive trait. In humans the *ADSL* gene spans approximately 23 kb on chromosome 22 (22q13.1q13.2) (Van Keuren et al 1987; Fon et al 1993). It consists of 13 exons and its promoter has typical features of house-keeping gene. The ADSL gene is transcribed in most of the tissues into two transcript variants which are produced by alternative splicing of exon 12. Full length variant encodes an active ADSL protein composed of 484 amino acids. The alternatively spliced variant encodes variant missing 59 amino acid (residues 397–456). This variant is catalytically inactive and its biological relevance is not clear yet (Kmoch et al 2000).

To date, over 50 different *ADSL* mutations have been identified in individuals with ADSL deficiency. A majority of the identified mutations represent missense mutations presenting in heterozygosis and producing an altered ADSL protein (Human Gene Mutation Database). A mutation of a regulatory region of the *ADSL* gene (in a nuclear respiratory factor 2 binding site in the promoter region) has also been identified in five patients (Marie et al 2002) as well as a mutation creating a new splice site and resulting in a 39 base pair deletion (Kohler et al 1999; Marie et al 1999). Mutation c.774_778insG results in the introduction of an early stop codon in position 284 of the protein (Chen et al 2010) and mutation c.889_891dupAAT predicted a duplication of the Asn297 residue at the active site of the enzyme (van Werkhoven et al 2013). Two-thirds of the diagnosed patients are compound heterozygotes and most mutations are private. The most common mutation, accounting for about one third of the patients’ alleles investigated, is a missense mutation R426H mutation.

## Purinosome

Recently, the existence of the purinosome has been described (An et al 2008). The purinosome is a multienzyme complex of the de novo purine synthesis (DNPS) enzymes (including ADSL) that cells transiently assemble in their cytosol upon depletion or increased demand of purines. The process of purinosome formation was first demonstrated and studied in HeLa and human C3A liver carcinoma cells transiently expressing recombinant fluorescently labeled DNPS proteins (An et al 2008). Then the existence of purinosome was proved in vivo conditions in cancer and normal non-transfected cells (Baresova et al 2012).

The later studies showed that the first three enzymes in the pathway form a core of the purinosome (Deng et al 2012) and are substrates for protein kinase CK2, which suggests dynamic regulation by post-translational modifications (An et al 2010a, b). The assembly and disassembly of purinosomes are correlated with the rate of DNPS and for its formation the intact network of microtubules is needed (An et al 2010a, b). Also methenyltetrahydrofolate synthetase association with the purinosome in mammalian cells was studied and dependence of this process on methenyltetrahydrofolate synthetase stimulation was observed (Field et al 2011).

The study of purinosome formation in skin fibroblasts of the patients with ADSL deficiency showed that significant differences of purinosome assembly exists among individual cases with ADSL deficiency and that the ability to form purinosomes inversely correlates with the severity of the phenotype (Baresova et al 2012). This finding corroborates the hypothesis that the phenotypic severity of ADSL deficiency is mainly determined by structural stability and residual catalytic capacity of the corresponding mutant ADSL protein complex, as this is a prerequisite for the formation and stability of the purinosome and at least partial channeling of SAICAR through the DNPS pathway (Zikanova et al 2010; Vliet et al 2011).

## Population variability of ADSL

Population sequencing (Exome sequence project) so far revealed 42 non-synonymous variants in ADSL gene, 25 is classified as probably damaging, four possibly damaging and 13 benign. Out of these variants five were already identified in patients, the most common mutation in ADSL patients R426H was not present. The probability of heterozygotes for a pathogenic *ADSL* mutation is about 1:10000.

## Genotype-phenotype correlations and structural stability

Current findings indicate that the severity of the clinical symptoms correlate with the residual enzymatic activity and stability of the recombinant mutant enzyme (Kmoch et al 2000; Ariyananda Lde et al 2009; Zikanova et al 2010; Ray et al 2013). In other words, the phenotypic severity in ADSL deficiency probably reflects the degree of structural stability and residual enzyme activity of the corresponding ADSL enzyme complex. This can be documented best by the biochemical properties of the recombinant mutant proteins associated with the neonatal fatal phenotype. These proteins display the lowest residual activity and significant thermal instability and the mutations mostly affect residues forming the catalytic sites or residues involved in substrate channeling. Recombinant mutant proteins associated with type II disease displayed mostly normal enzymatic activity; the proteins are stable, and the mutations seemed to be structurally benign based on *in silico* analysis of the ADSL structure. The pathogenic effects of these biochemically benign and structurally stable mutations, therefore, are probably not related to their intrinsic structural and/or catalytic properties but rather to abnormalities that may manifest only under *in vivo* conditions inside eukaryotic cells. Such mutations may affect protein folding, purinosome formation (Baresova et al 2012), slow down enzyme complex formation and/or lead to accelerated degradation. These mechanisms may decrease the amount of otherwise normally functioning enzyme complex and lead to a much milder enzyme deficiency (Zikanova et al 2010).

In all cases studied so far, the combination of mutations produce ADSL enzymes that retain some residual enzyme activity (Van den Bergh et al 1993a, b; Van den Bergh et al 1993a, b); complete lack of ADSL activity in humans is probably lethal.

## Pathophysiologic mechanisms

Hypotheses regarding the pathogenesis include toxicity of high levels of SAICAR, AMPS, or their metabolites, deficiency of the *de novo* purine biosynthetic pathway, or lack of a completely functional purine cycle in muscle and brain.

### Toxic effects of intermediates

The main pathogenic effect has been attributed to the toxic effects of accumulating succinylpurines (Stone et al 1998). Although the absolute S-Ado and SAICAr concentrations in body fluids do not correlate with the severity of the phenotype, it has been found that different values for the ratio between S-Ado and SAICAr concentrations in cerebrospinal fluid (S-Ado/SAICAr ratio) correspond with the three main phenotypic groups (Van den Bergh et al 1993a, b; Mouchegh et al 2007):

i) In patients with the neonatal fatal form, the S-Ado/SAICAr ratio is less than 1.
ii) In patients with the severe childhood form, the S-Ado/SAICAr ratio is close to 1.
iii) In patients with the moderate or mild form, the S-Ado/SAICAr ratio is more than 2.

The observation of less severe intellectual impairment in patients with similar SAICAr levels but S-Ado/SAICA-r ratios above 2, suggests that SAICA-r is the offending/neurotoxic compound, and that S-Ado may be protective of SAICAr’s effects. The finding that infusion of SAICAr to rats induces neuronal damage in specific regions of the hippocampus is consistent with this hypothesis (Stone et al 1998). To date, however, all attempts to demonstrate evidence of neurotoxicity of the succinylpurines in humans have failed.

Biochemical and structural analysis of mutant ADSL enzyme complexes have shown that different S-Ado/SAICAr ratios do not result from disproportionate SAMP and SAICAR enzyme activities. Almost all of the mutant protein complexes, if active, displayed a proportional decrease in activity toward both of the enzyme substrates.

It seems that the ratio of the accumulating S-Ado and SAICAr in body fluids is not predictive of phenotype severity; rather, it may be secondary to the degree of the patient’s development (i.e., to the age of the patient at the time of a sample collection) (Zikanova et al 2010).

### Deficiency of Purine Nucleotides

Deficient synthesis of purine nucleotides caused by ADSL deficiency has been hypothesized to have detrimental effects during embryo development, primarily depending on *de novo* purine synthesis. However, normal levels of purine nucleotides were measured in various patients’ tissues (Van den Berghe and Jaeken 1986). This suggests that ADSL activity, although deeply reduced, may not be limiting for the *de novo* synthesis or that salvage pathway involving the enzymes hypoxanthine-guanine phosphoribosyltransferase, adenine phosphoribosyltransferase and adenosine kinase may compensate for the deficiency (Jaeken and Van den Berghe 1984; Jaeken et al 1988). Nevertheless, a deficiency of purine nucleotides could occur in some hitherto unidentified cell types with profound deficiency of adenylosuccinate lyase and low activity of the salvage pathway. Little is known about the actual purine levels in the living brain and about the regulation of purine synthesis during embryogenesis, which might be affected by deficits in purine metabolism.

### Impairment of energy metabolism

ADSL also participates in the purine nucleotide cycle along with AMP deaminase and adenylosuccinate synthetase, and the impairment of this cycle in ADSL deficiency has also been suggested to cause the disorder. Purine nucleotide cycle controls the level of fumarate, a Krebs cycle intermediate, and of AMP, particularly maintaining the ATP/AMP ratio in muscles (Van den Berghe et al 1992).

## Diagnosis, diagnostic criteria, diagnostic methods

### Diagnosis requires the following:

- demonstration of succinylaminoimidazolecarboxamide riboside (SAICAr) and succinyladenosine (S-Ado) in extracellular fluids such as plasma, cerebrospinal fluid and/or urine using HPLC with UV detection or HPLC-MS,
- mutation analysis — genomic and/or cDNA sequencing of *ADSL* gene and characterization of mutant proteins.

Diagnosis is supported by the following:

- evidence of clinical phenotype,
- demonstration of SAICAr and S-Ado in extracellular fluids such as urine, cerebrospinal fluid and/or plasma using simple screening tests such as Bratton-Marshall or thin-layer chromatography,
- the analysis of ADSL enzyme activity in cultured skin fibroblasts at an accredited laboratory to demonstrate large decrease of ADSL activity. Although ADSL enzyme activity levels may be different between testing laboratories, ADSL activity in diagnosed ADSL patients is generally between 2-20 % of the lower limit of normal ADSL activity (Salerno et al 1995). Enzyme assay in lysates is not completely reliable due to tissue heterogeneity of ADSL defect (Mouchegh et al 2007).

### Biochemical diagnostic methods

All standard laboratory investigations are normal: no signs of hypoglycemia, hypomagnesaemia or hypocalcaemia, and the laboratory metabolic investigations such as liver function tests, plasma lactate and pyruvate, ceruloplasmin, thyroid-stimulating hormone, amino acids, and organic acids are normal.

The deficiency of ADSL activity results in accumulation of SAICAR and SAMP in the cells and the presence of enormously elevated concentrations of their dephosphorylated forms, SAICAr and S-Ado in extracellular fluids — urine and CSF, and to a lesser extent also in plasma. SAICAr and SAdo are present in low micromolar concentrations in body fluids of controls (Sebesta et al 1997; Krijt et al 1999). Several methods have been described for selective screening of subjects with ADSL deficiency that allow identification of either two or one of the relevant compounds in body fluids. The most commonly used include Bratton-Marshall test, thin-layer chromatography (TLC) for identification of SAICAr (Wadman, de Bree et al 1986), S-Ado (Maddocks and Reed 1989) and both nucleosides (Jaeken and van den Berghe 1989), isolation of SAICA riboside and S-Ado with a cation exchange resin and determination of A_270_/A_250_ ratio (UV absorbance of the ammonia eluate at 270 and 250 nm) (Domkin et al 1995), capillary electrophoresis (Gross et al 1995; Hornik et al 2007), and high resolution proton magnetic resonance spectroscopy (Wevers et al 1995; Henneke et al 2010). Bratton-Marshall test and TLC with Pauly reagent detects the presence of excessive urinary SAICA riboside (Laikind et al 1986), but may be false negative due to bacteria-mediated deribosylation of SAICAr and SAdo in urine (Krijt et al 2013) or SAICAr instability. The advantage of the Bratton-Marshall test is its simplicity of equipment and procedure, and the speed of test (Laikind et al 1986). HPLC-DAD, LC-MS/MS or other screening methods allowing simultaneous detection of SAICA-riboside ade and SAdo should be preferentially used for the diagnosis of ADSL deficiency. HPLC-DAD method employs resolution of SAdo and SAICAr from serum, urine, and CSF by reverse-phase high-pressure liquid chromatography (RP-HPLC) with detection by UV spectroscopy (Jaeken and Van den Berghe 1984; de Bree et al 1986; Jaeken et al 1988). The compounds are identified by spectral analyses and comparison to available standards. This method gives accurate results and has the advantage of quantifying the excretion of a large number of compounds. However, it requires expensive, specialized equipment and is usually employed as a second step to further identify the metabolite accumulating in any sample that gives a positive Bratton-Marshall or TLC test. Recently, high-throughput urine screening techniques for ADSL deficiency have been developed using HPLC combined with electrospray ionization (ESI) tandem mass spectrometry (MS/MS) (Ito et al 2000; Hartmann et al 2006; van van Werkhoven et al 2013). These methods allow rapid and specific screening for disorders of purine and pyrimidine metabolism with use of liquid urine samples or urine-soaked filter paper strips.

## Differential diagnoses

Differential diagnoses within ADSL deficiency include:

Neurological disorders with intractable seizures and encephalopathy

Other inborn errors of purine and pyrimidine (P/P) metabolism with neurological manifestations

The wide clinical spectrum of the disease accounts for possible difficulties in differential diagnosis with neurological disorders especially those with intractable seizures and encephalopathy.

In the neonatal period, differential diagnosis should firstly be made with five treatable disorders that can present predominantly with intractable seizures: pyridoxine responsive seizures, pyridox(amine)-5'-phosphate oxidase deficiency, folinic acid-responsive seizures, 3-phosphoglycerate dehydrogenase deficiency, and hyperinsulinemic hypoglycemia (Saudubray et al 2006). Biotin-responsive holocarboxylase synthetase deficiency can also rarely present with neonatal seizures. In the first months of life, biotin-responsive biotinidase deficiency and GLUT1 deficiency, treatable by a hyperketotic diet, can also present with intractable seizures. Other, nontreatable inborn errors of metabolism can present in the neonatal period with severe epilepsy: nonketotic hyperglycinemia, D-2-hydroxyglutaric aciduria, mitochondrial glutamate transporter defect, peroxisomal biogenesis defects, respiratory chain disorders, sulfite oxidase deficiency, and Menkes disease. In infancy, the association of seizures and autistic features should prompt differential diagnosis with Angelman syndrome.

Other inborn errors of purine and pyrimidine metabolism may present early in life with similar symptoms as ADSL deficiency. Selective screening for purines and pyrimidines is very valuable especially in children with nonspecific neurological symptoms.

## Prenatal diagnosis, selective screening and genetic counseling

Prenatal diagnosis is limited to families having a previous child with ADSL deficiency and is based on mutational analysis for at-risk fetuses. Diagnostic testing may be conducted for prenatal diagnosis on viable fetal cells from chorionic villi, cultured amniotic fluid cells or in the newborn dried blood spots (Marie et al 2000).

### Selective screening for ADSL deficiency

The wide clinical spectrum of the disease accounts for possible difficulties in differential diagnosis with neurological disorders especially those with intractable seizures and encephalopathy. For this reason, it is important to develop protocols for ADSL suspects and diagnosis to avoid useless investigations and treatments. The marked clinical heterogeneity justifies systemic screening for the disorder in patients with:

- newborns and infants with hypotonia and acquired microcephaly,
- unexplained psychomotor retardation,
- unexplained developmental delay,
- unexplained seizures especially intractable,
- MRI findings such as atrophy of the cerebral cortex, corpus callosum, cerebellar vermis, lack of myelination, delayed myelination, anomalies of the white matter.

## Specialties involved

Greater awareness of adenylosuccinate lyase deficiency amongst pediatricians, neonatologists, pediatric neurologists but also radiologists is the key to identifying the disorder in the early stage. Because the majority of newborns with neonatal as well as type I of ADSL deficiency have microcephaly, general pediatricians and neonatologists should exclude ADSL in newborns and infants with a small head circumference. General and emergency care pediatricians must consider ADSL deficiency in parallel with status epilepticus. Pediatric neurologists have an important role to play in bringing this disorder to the attention of the broader pediatric community and instructing them as to the key diagnostic clues. The finding of atrophy of the cerebral cortex, corpus callosum, cerebellar vermis, lack of/or delayed myelination, anomalies of the white matter or lissencephaly on MRI examination should alert neonatologists and radiologists to the possibility of ADSL deficiency.

With such an approach, more patients may benefit from early diagnosis.

### Genetic counseling

When the prospects for treatment or disease-altering management are not prominent, patients and family members will benefit from definitive diagnosis. Diagnosis helps the patient and family members gain access to appropriate organizations that provide emotional support as well as practical advice and assistance in dealing with the life changes that arise as a result of disability. Accurate diagnosis is vital so that families can receive genetic counseling about risk recurrence and the possibility of a- or pre-symptomatic affected relatives.

## Therapeutic approaches

### D-ribose and uridine administration

Despite the increasing number of ADSL-deficient patients reported, there are only a few communications of therapeutic considerations and efforts. Among them only two showed some beneficial effects (D-ribose and uridine administration) (Salerno et al 1998; Salerno et al 2000). D-ribose administration, which increases the provision of phosporibosylpyrophosphate (PRPP) and stimulates *de novo* purine synthesis, has been applied in a few ADSL patients. Salerno et al published promising results in motor coordination and seizure control in a 13-year-old female after several months of D-ribose therapy (Salerno et al 1998). These results, however, have not been confirmed in further studies (Jurecka et al 2008a, b; Perez-Duenas et al 2011).

#### S-adenosyl-l-methionine

Recently Werkhoven et al evaluated S-adenosyl-l-methionine (SAMe) as a potential treatment for ADSL deficiency (van Werkhoven et al 2013). After 9 months of SAMe treatment, there was no clear response evidenced in urine metabolite levels or clinical parameters.

#### Epilepsy treatment

The aim of treating epilepsy is to control or at least decrease seizure frequency with minimal side effects. Treatment with anticonvulsive drugs (e.g., valproic acid, phenobarbital, carbamazepine, topiramate, levetiracetam, phenitoin, clobazam) depends on the type of seizures. Patients with ADSL deficiency often require polypharmacy with the use of two or more anticonvulsants. Drug resistance is common.

#### Ketogenic diet

Recently there have been reports about positive use of a ketogenic diet for treatment of refractory epilepsy (Lefevre and Aronson 2000; Jurecka et al 2012a, b). Physiological consequences of a ketogenic diet are similar to fasting and include hypoglycemia and increased levels of ketone bodies and fatty acids. Various mechanisms have been hypothesized for the anticonvulsant effect of the ketogenic diet in ADSL patient (Jurecka et al 2012a, b). Some of these hypotheses focus on ketone bodies as crucial mediators of the beneficial effects of the ketogenic diet. Acetone shows the best anticonvulsant effects both *in vivo* and *in vitro* models (Masino and Geiger 2008). As a result of the diet, more glutamate becomes accessible to the glutamate decarboxylase reaction to yield gamma-aminobutyric acid (GABA), the major inhibitory neurotransmitter and an important antiseizure agent. In addition, the ketogenic diet appears to favor the synthesis of glutamine, an essential precursor to GABA (Yudkoff et al 2007). Both hypoglycemia and decreased pH are physiological changes that have been shown to increase adenosine and ATP levels. A purinenergic signaling hypothesis assumes neuroprotective and antiseizure actions of a ketogenic diet. Whilst on a ketogenic diet, increased mitochondrial biogenesis, brain energetics and brain ATP are observed.

A ketogenic diet could be considered a valid therapeutic option in patients with intractable seizures in a course of ADSL deficiency, however due to the possibility of side effects that may present during the diet, patients require a thorough and systematic examinations, including biochemical parameters of blood (Jurecka et al 2012a, b).

## Prognosis

Lifespan in patients with adenylosuccinate lyase deficiency is variable: neonatal forms may lead to early death, whereas onset in early childhood usually entails stable evolution. There are important issues to be considered when counseling families regarding prognosis and prenatal diagnosis. One is uncertainty regarding the long-term outcome in patients with milder phenotypes. Another complicating issue in genetic counseling is the unpredictability of phenotype in individuals with the same ADSL phenotype. The clinical heterogeneity in ADSL phenotype is not well understood.

## Concluding remarks

Adenylosuccinate lyase deficiency is a progressive disease with central nervous system involvement. As our knowledge about the natural history of ADSL deficiency increases, it becomes evident that the disease manifests symptoms along a continuum ranging from severe to mild. Clinical heterogeneity is especially prominent in the less severe end of the spectrum and some attenuated patients may present no obvious signs of disease progression and degradation. Case reports/series are therefore particularly important to expand our knowledge and understanding of the disease. The pathomechanism of the disease still remains unclear and requires further research. To date, there is no specific and effective therapy for adenylosuccinase deficiency despite a few attempts that have been made. The purinosome may also represent a new pharmacological opportunity for therapeutic intervention.

## Abbreviations

ADSL: adenylosuccinate lyase deficiency
AICAR: aminoimidazole carboxamide ribotide
CSF: cerebrospinal fluid
HPLC: high-pressure liquid chromatography
MRI: magnetic resonance imaging
MS/MS: tandem mass spectrometry
PP: purine and pyrimidine
SAICAR: succinylaminoimidazole carboxamide ribotide
SAICAr: succinylaminoimidazole carboxamide riboside
S-Ado: succinyladenosine
TLC: thin layer chromatography
